# *Tylosema esculentum* (Marama) Tuber and Bean Extracts Are Strong Antiviral Agents against Rotavirus Infection

**DOI:** 10.1155/2011/284795

**Published:** 2011-02-20

**Authors:** Walter Chingwaru, Runner T. Majinda, Sam O. Yeboah, Jose C. Jackson, Petrina T. Kapewangolo, Martha Kandawa-Schulz, Avrelija Cencic

**Affiliations:** ^1^Department of Microbiology, Biochemistry, Molecular Biology and Biotechnology, Faculty of Agriculture and Life Sciences, University of Maribor, Pivola 10, 2311 Hoče, Slovenia; ^2^Department of Chemistry, University of Botswana, Private Bag 0022 Gaborone, Botswana; ^3^Research and Development Office, University of Botswana, Private Bag 0022 Gaborone, Botswana; ^4^Department of Chemistry and Biochemistry, University of Namibia, 340 Mandume Ndemufayo Avenue Pionierspark, Private Bag 13301 Windhoek, Namibia; ^5^Faculty of Medicine, University of Maribor, Slomškov trg 15, 2000 Maribor, Slovenia

## Abstract

*Tylosema esculentum* (marama) beans and tubers are used as food, and traditional medicine against diarrhoea in Southern Africa. Rotaviruses (RVs) are a major cause of diarrhoea among infants, young children, immunocompromised people, and domesticated animals. Our work is first to determine anti-RV activity of marama bean and tuber ethanol and water extracts; in this case on intestinal enterocyte cells of human infant (H4), adult pig (CLAB) and adult bovine (CIEB) origin. Marama cotyledon ethanolic extract (MCE) and cotyledon water extract (MCW) without RV were not cytotoxic to all cells tested, while seed coat and tuber extracts showed variable levels of cytotoxicity. Marama cotyledon ethanolic and water extracts (MCE and MCW, resp.) (≥0.1 mg/mL), seed coat extract (MSCE) and seed coat water extract (MSCW) (0.01 to 0.001 mg/mL), especially ethanolic, significantly increased cell survival and enhanced survival to cytopathic effects of RV by at least 100% after in vitro co- and pre-incubation treatments. All marama extracts used significantly enhanced nitric oxide release from H4 cells and enhanced TER (Ω/cm^2^) of enterocyte barriers after coincubation with RV. Marama cotyledon and seed coat extracts inhibited virion infectivity possibly through interference with replication due to accumulation of nitric oxide. Marama extracts are therefore promising microbicides against RV.

## 1. Introduction

*Tylosema esculentum* (Burch.) (marama) A. Schreib. (family Caesalpiniaceae or Leguminosae) [1], also known as “the Green Gold of Africa,” is a creeping plant found in the southern parts of Africa, namely South Africa, Namibia, and Botswana*. Tylosema esculentum *bean and tuber extracts have been used in traditional African medicine to treat diarrhoea and for general upkeep of human health [2]. Only little chemical characterization has been done to date on the *T. esculentum *plant. Research on chemical and health benefits of *T. esculentum *plant is part of an ongoing research project under EU-INCO Marama II FP6 programme (contract number 032059). 

Various bioactive constituents are expected to be present in *T. esculentum *plant. These may include phenolic constituents, carbohydrates, and certain fatty acids, among many components. Large amounts of gallic acid have been detected in *T. esculentum *plant [3]. Gallic acid esterifies with glucose, the resultant hydrolysable tannins (HTs), secondary metabolites widely distributed in the plant kingdom, are known to be effective antagonists against viruses [3]. *Tylosema esculentum *tubers contain yet to be determined phenolics [3]. High amounts of phytosterols have also been detected in *T. esculentum *oil (about 75% of all phytosterols being 4-desmethylsterols and about 15.72% of the total being 4,4-dimethylsterols) [4].

Rotavirus (RV) infections are a major cause of acute gastroenteritis in infants and young children, accounting for about 611,000 deaths each year worldwide, mostly in developing countries [5]. Rotavirus is also an important pathogen in many agricultural species, inducing serious diarrhoeal diseases in neonatal and postweaning pigs and calves [6–8]. Rotavirus infection is limited to the mature enterocytes of the tips of the intestinal villi of humans, domestic animals, and mice, leading to severe gastroenteritis in the young [9]. Some strains of bovine and human RV have high sequence homology and interspecific infectivity, both implying phylogenetic relatedness [10, 11]. Previous *in vitro* tests of the bovine RF strain of RV on human, pig, chicken, and goat intestine epithelial cells in our laboratory have shown interspecific infectivity of rotavirus [12], and, therefore, the bovine strain of rotavirus was used to infect human and pig intestinal cells in the current study.

It is known that infection of intestinal epithelial cells with RV causes disruption of tight junctions and loss of transepithelial resistance (TER), even in the absence of cell death [13]. Loss of TER involves alteration of tight junction proteins, mainly claudin-1, occludin, and ZO-1 as determined in human small intestine carcinoma cells (Caco2) [13], although use of cancer cells for such mechanistic studies is not appropriate. Rotavirus infection is also associated with increased production of lactate, decreased mitochondrial oxygen consumption, and reduced cellular ATP, conditions known to reduce the integrity of epithelial tight junctions [13, 14]. 

While vaccines such as RataTeq and Rotatrix have been made available for prevention of RV infections [15, 16], their effectiveness remains to be verified [17]. Use of a few synthetic compounds against simian RV, such as ribavirin [18] and isoprinosine [19], and natural products against human and bovine RV [20], such as theaflavins, has been reported. Unfortunately, these compounds are not available for human use, which necessitates alternative methods to control RV infection [21]. A promising alternative strategy to reducing the burden of diarrhoea caused by RV may lie in identifying and developing cost-effective nutritional or phytomedical solutions; which can be applicable especially in children and immunocompromised persons. 

**Figure 1:**
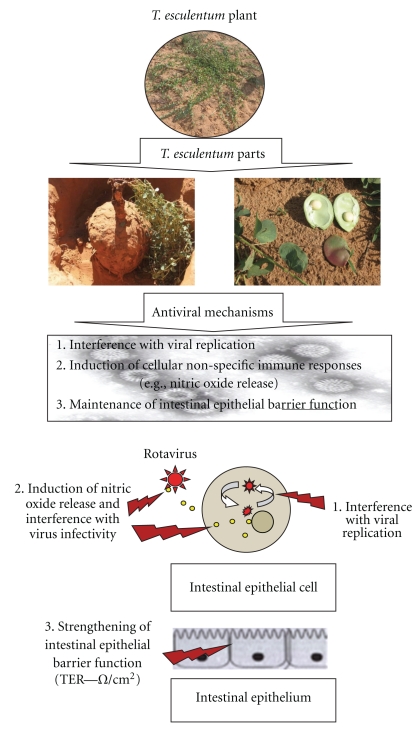
Proposed mechanisms of antirotaviral activity of *T. esculentum *plant parts.

With this information, and related empirical supporting information from other legume plants, it was hypothesised that *T. esculentum *bean and tuber extracts can inhibit RV infection. The proposed mechanisms of antiviral effects of *T. esculentum *are summarised in Figure 1. It was herein projected that extracts from *T. esculentum *beans and tubers can be included in various medicine mixtures or whole beans and tubers be used as functional foods, with the aim to combat diarrhoea caused by RV. 

In this study we sought to examine the antirotaviral activity of water and ethanol extracts from bean cotyledons and bean seed coats and water extracts from *T. esculentum *tubers by investigating their ability to increase survival of human foetal, pig, and calf intestinal epithelial cells. 

## 2. Materials and Methods

### 2.1. Collection of T. esculentum Bean and Tuber Samples

All research on the plant was done with full authorisation granted to Marama II INCO (EU-FP6) research programme. *T. esculentum *(marama) bean samples were obtained in Botswana in 2005 and 2007, and the *T. esculentum *tuber sample was obtained in Botswana in 2007. Seeds were stored in plastic bags at room temperature until time for extraction. The *T. esculentum *tuber (approximately 20 kg wet mass) was dug out from a site in Jwaneng, then ground and air-dried at University of Botswana, and then further ground to fine powder before the extraction was done at University of Maribor, Slovenia. A voucher specimen of *T. esculentum* plant is kept in the Namibian National Herbarium (collecting number: GM 1063 and herbarium number: 59520).

### 2.2. Extraction of T. esculentum

#### 2.2.1. Water Extraction

Shade-dried bean and tuber materials (20 g each) were ground to fine powder, the volume was adjusted to 200 mL by addition of distilled water, and left for 16 hours at room temperature. The mixture was blended to mix and then centrifuged at 4000 rpm (2050 g) for 30 minutes using a Sorval Evolution RC Superspeed centrifuge (Thermo, USA) at 4°C. The supernatant was filtered using 0.45 *μ*m pore filters (Millipore, Ireland) and then stored at −20°C until use. The *T. esculentum *water extracts, namely, seed coat water extract (MSCW), cotyledon water extract (MCW), and tuber water extract (MTW), were evaporated with a Soxhlet apparatus (Carl Roth WHLG2/ER-Serie, Karlsruhe, Germany) and then stored at −20°C until use. The following water extracts from *T. esculentum *beans and tuber were obtained: tuber water extract (MTW), cotyledon water extract (MCW), and seed coat water extract (MSCW) (Table 1).

#### 2.2.2. Ethanolic Extraction Procedure

Crude bean ethanolic extracts were prepared as described by Bolling and Parkin [22]. Briefly, 1 litre of ethanol was used to extract 100 g of shade-dried *T. esculentum *bean at 78°C using a Soxhlet apparatus (Carl Roth WHLG2/ER-Serie, Karlsruhe, Germany) for extraction. The evaporation procedure ensured the exclusion of ethanol from the extracts; the resultant extract was a thick paste; these extracts were labelled MSCE (seed coat ethanolic extract) and MCE (cotyledon ethanolic extract) and stored at −20°C until use. Ethanolic extracts are known to contain mainly lipids and other ethanol soluble contents such as isoflavones [22–24]. The following extracts from *T. esculentum *beans and tuber were obtained: cotyledon ethanolic extract (MCE) and seed coat ethanolic extract (MSCE) (Table 1). 

Below are the yields of each extract (mg) per g of *T. esculentum *bean and tuber material used.

#### 2.2.3. Cells and Viral Strains

**Cell cultures**

The following cell lines were used in the antiviral tests: human foetal small intestine epithelial cells (H4) [25] (a generous gift from Dr. Tor Savidge, USA), pig small intestine epithelial cells (CLAB), isolated in our laboratory [26] and maintained by University of Maribor, Slovenia, and bovine calf small intestine epithelial cells (CIEB), isolated in our laboratory [26] and maintained by University of Maribor, Slovenia. The cells were grown in advanced Dulbecco's Modified Eagle's Medium (DMEM) (Sigma-Aldrich, Grand Island, USA), supplemented with 10% foetal calf serum (Cambrex, Verviers, Belgium), L-glutamine (2 mmol/L, Sigma), penicillin (100 units/mL, Sigma), and streptomycin (1 mg/mL, Fluka, Buchs, Switzerland). Cell lines were routinely grown in 25 cm^2^ culture flasks (Corning, New York, USA) at 37°C in a humidified atmosphere of 5% CO_2_ and 95% air until confluent monolayers attained. Culture medium was changed routinely.

#### 2.2.4. Virus Propagation

An RF RV strain, as previously described in [27, 28], was used in all tests. Confluent cultures (in 25 cm^2^ Corning flasks) of H4, CLAB, and CIEB cells were washed twice in single strength phosphate buffer (1  ×  PBS) to remove the foetal bovine serum (FBS) before infection with RV (previously passaged 5 times on H4, CLAB, and CIEB cells in rotation; at 1  ×  10^7.5^/mL TCID_50_ in the presence of 1 *μ*g of trypsin (Sigma Chemical Co., St. Louis, Mo) per mL. After a 1-hour adsorption period at 37°C, DMEM, containing 0.5% FBS (Sigma Chemical Co., St. Louis, Mo.) and 1 *μ*g of trypsin per mL, was added to each flask. The flasks with cells were incubated for 24 to 48 h at 37°C, under 10% CO_2_ until cytopathic effect (CPE) was observed by microscopy. When the cells showed extensive cytopathic degeneration, the flasks with cells and virus were subjected to two freeze-thaw cycles to detach and break the cells. The supernatant fluids containing detached cells and extracellular virus were removed, and then the cellular debris was removed by centrifugation at 3500 g for 10 min. Virus was stored at −70°C until time for tests. 

Tissue culture infective dose (TCID_50_) was determined using the method of Reed and Muench [29], as previously described in [30]. Clarified supernatants of RV titrating 1  ×  10^7.5^ TCID_50_/mL (tissue culture infectious dose 50%, TCID_50_) were subsequently used for the antiviral tests with *T. esculentum *extracts. *Similar TCID_50_ values were obtained by Bae et al.* [31]* (1.27  ×  10^6^ per mL) when they titrated *RV* on Macacus Rhesus monkey kidney cells (MA104).*

#### 2.2.5. Studies on Cytotoxicity of T. esculentum on Intestinal Epithelial Cells

*Tylosema esculentum *extracts were dissolved in DMEM (Sigma-Aldrich, Grand Island, USA), supplemented with 10% foetal calf serum (Cambrex, Verviers, Belgium), L-glutamine (2 mmol/L, Sigma), penicillin (100 units/mL, Sigma), and streptomycin (1 mg/mL, Fluka, Buchs, Switzerland) using a rotary magnetic stirrer (Yellowline, BigSquid, Kika-Werke GMBH & Co, Germany). As control, DMEM (Sigma-Aldrich, Grand Island, USA), supplemented with 10% foetal calf serum (Cambrex, Verviers, Belgium), L-glutamine (2 mmol/L, Sigma), penicillin (100 units/mL, Sigma), and streptomycin (1 mg/mL, Fluka, Buchs, Switzerland), was used. The extracts were added diluted 10-fold to monolayers of cells in 96 well plates, then maintained at 37°C in a humidified atmosphere of 5% CO_2_ and 95% air for 24 h. Following incubation, cells were carefully rinsed with PBS to remove *T. esculentum *extracts and other debris. Upon washing, plates were stained with 0.01% crystal violet for 5 minutes and then rinsed with water. Plates were then dried, and then crystal violet incorporated in viable cells was resuspended with 10% acetic acid (100 *μ*L per well). Photometric quantification of crystal violet previously retained in living cells was done at 595 nm with a microplate reader (Multiscan, Finland) as described by Agelis et al. [32]. After this test for cytotoxicity, certain concentrations of *T. esculentum *extracts were selected for antiviral tests as shown in Table 2.

#### 2.2.6. Studies on Protection of Intestinal Epithelial Cells against RV with T. esculentum Extracts

*Tylosema esculentum *ethanolic extracts, namely, seed coat ethanolic extract (MSCE) and cotyledon ethanolic extract (MCE) were dissolved using a rotary magnetic stirrer (Yellowline, BigSquid, Kika-Werke GMBH & Co, Germany) in DMEM (Sigma-Aldrich, Grand Island, USA), supplemented with 10% foetal calf serum (Cambrex, Verviers, Belgium), L-glutamine (2 mmol/L, Sigma), penicillin (100 units/mL, Sigma), and streptomycin (1 mg/mL, Fluka, Buchs, Switzerland). Rotavirus at 1  ×  10^7.5^/mL TCID_50_ was combined with concentrations of *T. esculentum *extracts (see Table 2). As controls, DMEM (Sigma-Aldrich, Grand Island, USA), supplemented with 10% foetal calf serum (Cambrex, Verviers, Belgium), L-glutamine (2 mmol/L, Sigma), penicillin (100 units/mL, Sigma), and streptomycin (1 mg/mL, Fluka, Buchs, Switzerland), dilutions of Combivir GlaxoSmithKline—150 mg lamivudine and 300 mg zidovudine (AZT) as antiviral agent as described by Agelis et al. [32, 33] and *T. esculentum *extract at below IC_50_ concentrations and RV solutions at 1  ×  10^7.5^/mL TCID_50_ were separately used. The plant extract and virus, Combivir, and virus mixtures were added to monolayers of cells in 96-well plates and then maintained at 37°C in atmosphere of 5% CO_2_ for 24 h. 

Cytopathic effects of RV alone and in coculture with *T. esculentum *extracts or Combivir added at 0.75 mg/mL {modified from Agelis et al. [33]} on cell monolayers after treatment as listed above were measured after 24-hour coincubation of cell monolayers (1  ×  10^6^ cells/mL) at 37°C in atmosphere of 5% CO_2_. It was previously shown that *T. esculentum *extracts alone were not cytotoxic at the concentrations used in these tests; hence the observed reduction in survival of cells upon coincubation of extract RV solutions was attributed mainly to the virus. Following incubation, relative viability of cells was determined photometrically in terms of amount of crystal violet incorporated in viable cells as described previously.

#### 2.2.7. Studies on Effect of Coincubation of Rotavirus with T. esculentum Extracts on Monolayer Polarity (TER) (%  Ω) of Human Small Intestine Epithelial Cells (H4) over Time

Human small intestinal epithelial cells (H4) were introduced into inserts in 12-well plates (Corning Transwell, 0.4 *μ*m pore size, New York, USA) at concentration of 1  ×  10^6^ cells/mL. Transepithelial resistance (TER—Ω/cm^2^) was measured periodically using Millicell-ERS Electrical Resistance System (Millipore, Bedford, USA) until values of about 1000 Ω/cm^2^ were attained, such high values indicating maximum attainable polarity in H4 cells, as previously determined (results not shown). Rotavirus suspensions (at 1  ×  10^7.5^/mL TCID_50_) were mixed with *T. esculentum *extracts at below their predetermined IC_50_ concentrations (Table 2) and then introduced onto H4 monolayers in insert wells. *Tylosema esculentum *extracts diluted in DMEM, DMEM alone, and Combivir diluted in DMEM were introduced as respective controls. The effect of the extracts on the cell polarity was evaluated by measurement of transepithelial electrical resistance (TER) as previously described in [34]. TER values of monolayers exposed to combinations of marama/Combivir treatments, DMEM, and RV controls were plotted.

#### 2.2.8. Inhibition of Virion Infectivity Studies

Rotavirus (at 1  ×  10^7.5^/mL TCID_50_) was incubated with *T. esculentum *water and ethanolic extracts at room temperature for 1 h. Following the incubation, dilutions of the incubated virus were added to CLAB, CIEB, and H4 cell monolayers in 96-well plates and then incubated for 1 h at 37°C in a humidified atmosphere of 5% CO_2_ and 95% air, after which the unattached virus and the extracts were washed off with PBS. DMEM (Sigma-Aldrich, Grand Island, USA), supplemented with 10% foetal calf serum (Cambrex, Verviers, Belgium), L-glutamine (2 mmol/L, Sigma), penicillin (100 units/mL, Sigma), and streptomycin (1 mg/mL, Fluka, Buchs, Switzerland), was then introduced on the cells and then incubated for 24 h at 37°C in atmosphere of 5% CO_2_ until viral cytopathic effects were observed in control wells. Cell viability was then determined with quantification of crystal violet as described previously. As control, viral only, *T. esculentum *only and cell culture medium only treatments were plated onto the epithelial cells. Relative protection by *T. esculentum *extracts against RV infection was determined by crystal violet staining. Percent (%) inhibition of RV by *T. esculentum *extracts as shown by survival of epithelial cells was calculated, thus

(*average OD of wells containing *RV* preincubated with T. esculentum extracts minus OD of wells containing *RV* only*) *divided by average OD of wells containing *RV* only multiplied by 100 *±%* average standard deviation. An indication of level of significance *(*)* as tested using Statsoft Statistica software *(*P* < .05)* was given. *

Tests were done in triplicate wells. *Tylosema esculentum *extracts used were as follows: cotyledon ethanolic extract (MCE), seed coat ethanolic extract (MSCE), tuber water extract (MTW), cotyledon water extract (MCW), and seed coat water extract (MSCW). *Tylosema esculentum *extracts were introduced at and below IC_50_ or at 1 and 0.1 mg/mL of MCE (the highest concentration of MCE was not cytotoxic).

#### 2.2.9. Total Nitric Oxide (NO) Release from H4 and CLAB Cells after Exposure to T. esculentum Extracts

Release of NO after cell exposure to *T. esculentum *extracts was detected using a microplate reader (Multiscan, Finland) at 540 nm after introduction of Griess reagent (Sigma, Germany) into supernatants after 24-hour incubation. Results were expressed as percent difference between nitric oxide release of test from control. Nitric oxide is proportional to the amount of nitrate accumulating in supernatants as shown by the change of colour at 540 nm.

#### 2.2.10. Correlation Testing

Correlation coefficients for data obtained on level of protection, nitric oxide release, and transepithelial resistance (TER) were calculated using Statsoft Statistica version 7 software.

## 3. Results

### 3.1. Rotavirus Has Dose-Dependent Cytopathic Effect on Intestinal Epithelial Cells

**Figure 2:**
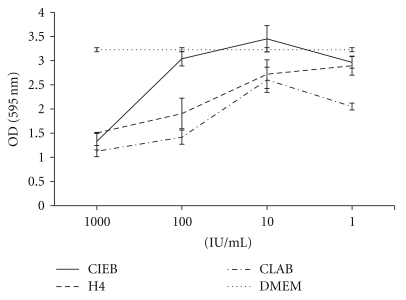
Dose-dependent cytopathic effect of rotavirus on bovine intestinal epithelial cells (CIEB), pig intestinal epithelial cells (CLAB), and human intestinal epithelial cells (H4). The graphs for cytopathic effect of rotavirus on the different cells are shown in terms of mean absorbance at each concentration ± standard deviation at 595 nm.

To determine concentration of RV in the stock solution, the method of Reed and Muench [29] was adopted with modifications. Titration results showed that the RV strain had high and variable but not cell-type-specific cytopathic effects on all the cells used (Figure 2). Rotavirus, when incubated without *T. esculentum *extracts on cells, showed dose-dependent cytopathic effect on human small intestine epithelial cells (H4 cells), pig small intestine epithelial cells (CLAB), and bovine calf small intestine epithelial cells (CIEB) (Figure 2). For subsequent antiviral testing an RV concentration at and/or below 1  ×  10^7.5^/mL TCID_50_ was used to avoid cotoxicity with extracts.

### 3.2. Tylosema esculentum Extracts Increase Survival of Intestinal Epithelial Cells

**Figure 3:**
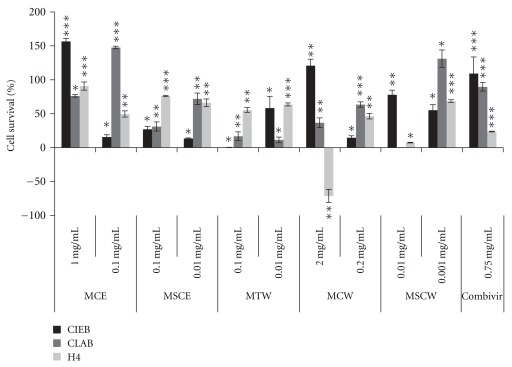
Survival of bovine intestinal epithelial cells (CIEB), pig intestinal epithelial cells (CLAB), and human intestinal epithelial cells (H4) from cytopathic effects of rotavirus in the presence of *T. esculentum *extracts. Shown above are bars of % survival of epithelial cells {% change in cell viability after coincubation of rotavirus with marama extracts compared to rotavirus only wells at OD 595 nm ±  % standard deviation}, respective levels of significance in terms of *T* values (*). *T* values were determined using Statsoft Statistica software (*P* < .05). Tests were done in triplicate wells. *T value between 0 and 5 (low level significance), **T value greater than 5, but less than 10 (middle level significance), and ***T value greater than 10 (high level significance). MCE:* T. esculentum* bean cotyledon ethanolic extract, MSCE:* T. esculentum* bean husk ethanolic extract, MTW:* T. esculentum* tuber water extract, MSCW:* T. esculentum* bean husk water extract, MCW: *T. esculentum* bean cotyledon water extract, and Combivir: (lamivudine and zidovudine).

*Tylosema esculentum *bean water and ethanolic extracts and *T. esculentum *tuber water extract were added on cell monolayers of CLAB, CIEB, and H4 cells at concentrations as shown in Table 2 and Figures 3 and 6. *Tylosema esculentum *extracts, at the concentrations used, resulted in dose-dependent enhancement of the three types of cells. Exposure to *Tylosema esculentum *seed coat water and ethanolic extracts (MSCW and MSCE, resp.) generally led to enhanced survival of CLAB, CIEB, and H4 cells. This result could show that exposure to cotyledon extracts, besides the projected inhibition of RV cytopathic effects, could ensure cell survival.

### 3.3. Tylosema esculentum Extracts Protect Intestinal Epithelial Cells from Cytopathic Effects of RV

*Tylosema esculentum *extracts had the highest protection against RV cytopathic effects on bovine intestinal epithelial (CIEB) followed by pig intestinal epithelial cells (CLAB) and lastly human intestinal epithelial cells (H4) as shown in Figure 3. All *T. esculentum *extracts, especially cotyledon ethanolic extract (MCE), led to significant protection of CIEB, CLAB, and H4 cells from cytopathic effects of rotavirus. *Tylosema esculentum *seed coat water extract (MSCW) offered significant protection at the lowest concentration (0.001 mg/mL), making it the most potent extract. *Tylosema esculentum *cotyledon water extract (MCW) also led to highly significant protection of CLAB cells. There was largely highly significant protection of H4 cells exposed to MSCE, MSCW, MTW, and MCE. Combivir (control) was shown to have highly significant protection of cells against RV. Exposure to MCE was shown to enhance survival of CIEB cells (56%) and H4 cells (275%) more than that to Combivir. On H4, there was higher protection of cells with all extracts (91%–275%) depending on concentration than Combivir alone (Figure 3). Some extracts, mainly seed coat and cotyledon water and ethanolic, led to higher protection at concentrations lower than the determined IC_50_.

### 3.4. Coincubation of Rotavirus with T. esculentum Extracts Results in Dynamic Effects on Monolayer Polarity (TER) (%  Ω/cm^2^) of H4 Cells over Time

**Figure 4:**
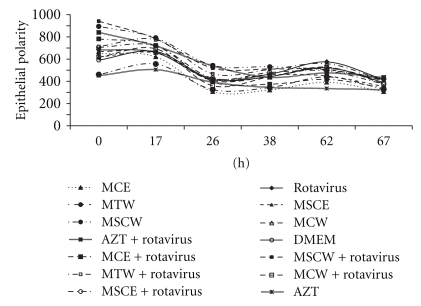
Effect of coincubation of rotavirus with *T. esculentum* extracts on monolayer polarity (TER) (Ω/cm^2^) of H4 cells over time. Shown graphs of change in epithelial cell monolayer polarity exposed to extracts or AZT and rotavirus (treatment wells), extracts or AZT alone (control), or rotavirus alone (negative control) over time compared to respective controls. Tests were done in triplicate wells (12-well Transwell plates). MCE:* T. esculentum* bean cotyledon ethanolic extract (1 mg/mL), MSCE:* T. esculentum* bean husk ethanolic extract (0.1 mg/mL), MTW:* T. esculentum* tuber water extract (0.1 mg/mL), MSCW:* T. esculentum* bean husk water extract (0.01 mg/mL), MCW:* T. esculentum* bean cotyledon water extract (2 mg/mL), and AZT: Combivir (lamivudine and zidovudine) (0.75 mg/mL).

Figure 4 shows changes in TER of H4 cells exposed to *T. esculentum *extracts, Combivir (AZT), or DMEM with and without RV over time. Over the whole 67 hours, coincubation of RV with bean seed coat extracts (ethanolic extract (MSCE), and water extract (MSCW) or AZT led to higher TER across the cells than when the virus was coincubated with cotyledon extracts (MCE and MCW). Incubation of H4 cells with individual extracts alone or AZT alone resulted in lower TER than extract/AZT-RV coincubation treatments. Incubation of cells with RV alone from the start of the experiment until about 40 hours resulted in TER lower than the rest of the treatments. However between 40 and 67 hours of exposure, wells with RV only treatments generally had higher TER (though sporadic) than the rest of the treatments. All *T. esculentum *extracts generally had better enhancement of TER than AZT over the initial 67 hours.

### 3.5. Exposure of Cells to T. esculentum Extracts Results in Variable Expression of NO from Human Intestinal Epithelial Cells (H4) and Pig Intestinal Epithelial Cells (CLAB)

**Figure 5:**
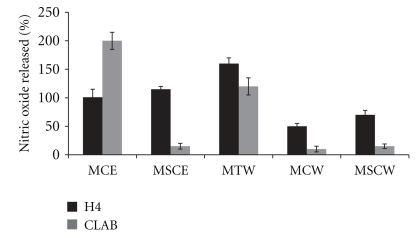
Effect of *T. esculentum* extracts on release of nitric oxide (NO) (% release compared to control) in human small intestine epithelial cells (H4) and pig small intestine epithelial cells (CLAB). MCE:* T. esculentum* bean cotyledon ethanolic extract (1 mg/mL), MSCE:* T. esculentum* bean husk ethanolic extract (0.1 mg/mL), MTW:* T. esculentum* tuber water extract (0.1 mg/mL), MSCW:* T. esculentum* bean husk water extract (0.01 mg/mL), and MCW:* T. esculentum* bean cotyledon water extract (2 mg/mL).

NO_2_ release was significant from human intestinal epithelial cells (H4) exposed to all extracts used {seed coat water and ethanolic extracts (MSCW and MSCE, resp.), cotyledon water and ethanolic extracts (MCW and MCE, resp.), and tuber water extract (MTW)}, while only MCE and MTW significantly increased NO in CLAB cells (Figure 5). 

### 3.6. Tylosema esculentum Extracts Inactivate Rotavirus

**Figure 6:**
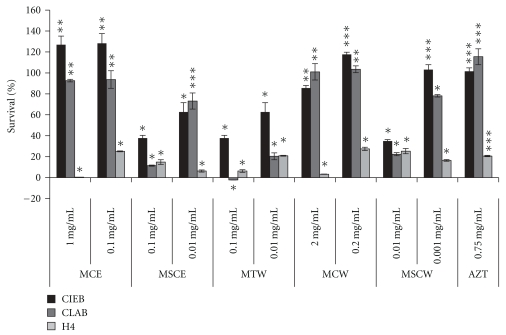
*T. esculentum *extracts show direct inhibition of rotavirus infectivity on bovine intestinal epithelial cells (CIEB), pig intestinal epithelial cells (CLAB), and human intestinal epithelial cells (H4). Shown above are bars of % survival of epithelial cells {% change in cell viability after pre-exposure of rotavirus to *T. esculentum *extracts compared to rotavirus only wells at OD 595 nm ±  % standard deviation}, respective levels of significance in terms of *T* values (*). *T* values were determined using Statsoft Statistica software (*P* < .05). Tests were done in triplicate wells. *T value between 0 and 5 (low level significance), **T value greater than 5, but less than 10 (middle level significance), and ***T value greater than 10 (high level significance). MCE:* T. esculentum* bean cotyledon ethanolic extract, MSCE:* T. esculentum* bean husk ethanolic extract, MTW:* T. esculentum* tuber water extract, MSCW:* T. esculentum* bean husk water extract, MCW:* T. esculentum* bean cotyledon water extract, and AZT: Combivir (lamivudine and zidovudine).

Results in Figure 6 show that pre-exposure to *T. esculentum *extracts can significantly reduce infectivity of RV. This finding broadly points to inhibition of RV cytopathic effect by *T. esculentum *extracts prior to or during virus entry (specific mechanisms were not determined). Exposure of RV (at 1  ×  10^7.5^/mL TCID_50_) to *T. esculentum *extracts *in vitro* resulted in species-specific and variable protection of the intestinal epithelial cells used. Pretreatment of RV with *T. esculentum *cotyledon ethanolic and water extracts (MCE and MCW) led to highly significant (***P ≥ 10) survival of CIEB cells, while pretreatment of virus with MSCE also resulted in highly significant survival of CLAB cells. While pretreatment of RV with all *T. esculentum *extracts resulted in significant enhancement of all cell types used (CLAB, CIEB, and H4 cells), *T. esculentum *seed coat water extract (MSCW) significantly reduced cytopathic effects of RV at the lowest concentration (0.001 mg/mL), implying high potency of this extract. Additionally other extracts significantly reduced cytopathic effects of RV, namely, *T. esculentum *cotyledon extracts (MCE 1 mg/mL and 0.1 mg/mL; MCW 2 mg/mL and 0.2 mg/mL). There was species-specific improvement in cell survival by the extracts, mainly bovine intestinal epithelial (CIEB) showed the highest response to pretreatment of virus with extracts.

### 3.7. Assessment of Correlation among T. esculentum and Antiviral Treatments

Table 3 shows correlation of antiviral effects of different treatments with *T. esculentum *extracts on different cell cultures. There was high positive correlation between CLAB and H4 cells (*r* =  0.94), moderately positive correlation between CLAB and CIEB cells (*r* = 0.57) in survival from rotavirus cytopathic effects after coincubation with *T. esculentum *extracts, while a weak negative correlation between H4 and CIEB cells (*r* = −0.02) was observed after coincubation. Highly positive correlation between survival of CLAB cells after coincubation compared to preincubation with *T. esculentum *extracts (*r* = 0.91) and moderately positive correlation between CIEB cells pre- and coincubated with the extracts (*r* = 0.59) were observed. Poorly positive correlation between H4 cells co- and pretreated with the extracts was observed. Moderate positive correlations were also observed between survival of and nitric oxide release from CLAB (*r* = 0.43) and H4 cells (*r* = 0.79), as well as highly positive correlation in levels of nitric oxide release from H4 and CLAB cells (*r* = 0.9). Negative correlation was observed between survival rates and TER of cells (H4, CLAB and CIEB) after coincubation of rotavirus with *T. esculentum* extracts. 

## 4. Discussion

We show herein that the RV strain used (RF strain) could be adapted to pig and human cells, additional to bovine cells. *T. esculentum* bean cotyledons had low or no cytotoxicity, as shown by high IC_50_ concentrations (Table 2; Figures 3 and 6), while seed coat and tuber extracts, especially seed coat ethanolic extract (MSCE) and tuber water extract (MTW), had elevated cytotoxic effects (low IC_50_ concentrations lying between 0.01 and 0.001 mg/mL) on all the cells used (Table 2; Figures 3 and 6). This study has identified *T. esculentum *bean ethanolic cotyledon extract (MCE) and bean seed coat extract (MSCE) as having highly significant inhibitory effects against cytopathic effects of RV, both with coincubation (Figure 3) and preincubation (Figure 6) of the extracts and RV on human, pig, and bovine intestinal epithelial cells. *Tylosema esculentum *seed coat ethanolic extract (MSCE) showed the best antirotaviral activity, as shown by the lowest IC_50_ concentrations (1 *μ*g/mL). 

Marama cotyledon ethanolic extract (MCE) offered similar and/or higher protection against cytopathic effects of RV than that offered by antiviral drug Combivir on all intestinal epithelial cells, with higher effect on CIEB and H4 cells. Exposure of CLAB cells to seed coat and cotyledon water and ethanolic extracts at concentrations lower than IC_50_ resulted in higher survival of the cells. This could be due to potentiated toxic effects due to combined presence of RV and extract (at IC_50_ concentration) and diluted cytotoxicity at lower extract concentrations. This effect was cell culture dependent, implying possible different interspecies responses. 

There was positive correlation between levels of protection of intestinal cells from cytopathic effects of RV (crystal violet assay) with coincubation and preincubation and enhancement of polarity (TER) of H4, CLAB, and CIEB cells by *T. esculentum *extracts and Combivir (Table 3). Survival of CLAB and H4 cells was moderately to highly correlated to release of nitric oxide (*r* = 0.79 and *r* = 0.43, resp.); similarly very high positive correlation was observed in levels of nitric oxide release from H4 and CLAB cells (*r* = 0.9) exposed to *T. esculentum *extracts (Table 3). These findings show that there is a positive link between effects of *T. esculentum *extracts (pre- and coincubated) against rotavirus on the different species of cells and the release of nitric oxide. 

Coincubation of RV and marama seed coat ethanolic extract (MSCE), cotyledon ethanolic extract (MCE), or tuber water extract (MTW) on H4 cells resulted in generally higher enhancement of TER than Combivir throughout 67-hour test period. Enhancement of polarity across intestinal epithelial cells can be an important mechanism to control entry of RV across the intestinal epithelial barrier. Rotaviruses are known to disrupt tight junctions, resulting in loss of transepithelial resistance (TER) [13], but without cell death during viral replication [35]; hence *T. esculentum *bean seed coat and cotyledon ethanolic extracts (MSCE and MCE, resp.) and *T. esculentum *tuber water extract (MTW) can reduce entry of RV across the intestinal barrier *in vitro. *Effects of *T. esculentum *extracts on transepithelial resistance of H4 cells, however, could not be linked to survival of H4 cells, as shown by negative correlation between survival of cells and TER in presence of the extracts. 

Another mechanism of inhibition of RV by *T. esculentum *extracts, especially in seed coats, may lie in the inactivation of RV, as shown with enhancement of cell survival after preincubation of rotavirus with *T. esculentum *extracts. It is herein suggested that the observed inactivation of RV may be through interference with either viral replication or capacity to bind to permissive cells. Clark et al. [36] have shown that some phenolic acids, which were expected to dominate the ethanolic extracts, have inactivation effect against RV. Similar observations were also made with extracts from peppermint, sage, and lemon balm leaves, which were highly inhibitive against HIV [37]. The inhibitive effects of *T. esculentum *extracts, especially seed coat and cotyledon ethanolic extract (MSCE and MCE) and tuber water extract (MTW) (≥100%), could additionally be due to enhanced release of nitric oxide (NO) from intestinal cells. Nitric oxide has been shown to inactivate RV, mainly by inhibiting replication of the virus [38, 39]. The observed enhancement of nitric oxide release from the human and pig cells was viewed as one of the mechanisms *T. esculentum *crude extracts can induce a nonspecific immune response in intestinal epithelium. 

Certain plant phytochemicals have been shown to inhibit RV activity through inhibition of viral penetration and viral replication. Some flavonoids were shown to inhibit penetration of RV in rhesus monkey epithelial cell line (MA104) [31], while others have an effect on the viruses or on the enzymes responsible for their replication [40]. These effects could not be singly demonstrated in this study; further work on these possible mechanisms of antiviral effects of *T. esculentum *extracts is, therefore, encouraged. 

The observed antirotaviral effects can be attributed to the high phenolic acid content (especially gallic acid), in bean cotyledons, among other components (Marama II report, *unpublished*). Gallic acid was previously shown to prevent viral replication, inhibition of virus attachment to and penetration into cells, and virucidal effects [41]. Interference with attachment and penetration into cells could not be proved in this study. *Tylosema esculentum *tubers may contain some yet to be determined phenolics (Marama II report, *unpublished*). Ethanolic extracts had notably higher antiviral activity than aqueous bean and tuber extracts (Figures 3 and 6). Some essential oils as found in *T. esculentum *beans may contribute towards the observed antiviral effects; similar findings were reported by Astani et al. [42] using essential oils extracted from *Melaleuca alternifolia*. Moreover, recently, a Kunitz-type inhibitor, distinct from other known plant serine protease inhibitors and shown to be specific for elastase, was isolated in *T. esculentum *beans [43]. The quantity of this elastase inhibitor present in *T. esculentum *beans is many times greater than in soybean or any other bean or nut source reported to date [43]. Certain protease inhibitors have been shown to impart high antienteroviral effects through interference with proteolytic cleavage during the replication process [44]. The high phytosterol content of *T. esculentum *bean oil 4-desmethylsterols (75%) and 4.4-dimethylsterols and 4-monomethylsterols (15.72%) [4] could also contribute to the high antiviral activity in bean extracts. Phytosterols have been determined to have antihuman cytomegalovirus (HCMV) and antiherpes simplex virus (HSV) effects [45]. The antiviral activity of phytosterols is through the blocking effect on immediate-early antigen expression in fibroblast cells and blocking of virus-cell interaction and/or virus multiplication [45]. 

Our results have shown high *in vitro *inhibition of RV by *T. esculentum *extracts, especially seed coat ethanolic extracts (0.01 to 0.001 mg/mL) and cotyledon ethanolic extracts (≥0.1 mg/mL), and tuber water extract (0.1 to 0.01 mg/mL). Mechanisms of antiviral action may include release of nitric oxide and may also interfere with viral cytopathic effects (as shown by moderate to high positive correlation between survival from rotavirus and release of nitric oxide), inactivation of RV (inhibition of virus replication or entry into cells may be responsible), possibly through interference with replication, and enhancement of tight junctions (though not directly related to levels of survival) (Figure 1). Our findings suggest that *T. esculentum *beans can be an important source of microbicides against RV. Further phytochemical characterization of *T. esculentum *extracts, identification of the responsible bioactive compounds, and the elucidation of other modes of action and quality standard studies are essential.

## Figures and Tables

**Table 1:** Yields of *T. esculentum* bean and tuber extracts (mg of extract per gram of plant material).

Extract	Yield (mg/g)
MCE	100
MSCE	100
MTW	26.19
MCW	19.55
MSCW	10.78

MCE: *T. esculentum* bean cotyledon ethanolic extract, MSCE:* T. esculentum* bean husk ethanolic extract, MTW:* T. esculentum* tuber water extract, MSCW:* T. esculentum* bean husk water extract, and MCW:* T. esculentum* bean cotyledon water extract.

**Table 2:** Selected concentrations of *Tylosema esculentum *extracts (IC_50_ and below).

Extract	Concentration of marama used in tests (mg/mL of solution)/(mg/g of plant material)
MCE	1/0.1
	0.1/0.01
MSCE	0.1/0.01
	0.01/0.001
MTW	0.1/0.01
	0.01/0.001
MCW	2/0.2
	0.2/0.02
MSCW	0.01/0.001
	0.001/0.0001

MCE:* T. esculentum* bean cotyledon ethanolic extract, MSCE:* T. esculentum* bean husk ethanolic extract, MTW:* T. esculentum* tuber water extract, MSCW:* T. esculentum* bean husk water extract, and MCW:* T. esculentum* bean cotyledon water extract.

**Table 3:** Correlation analysis of antiviral effects of different treatments with *T. esculentum *extracts on different cell cultures.

Treatment	Correlation coefficient (*r*)
(a) Survival of H4 cells compared to CLAB cells from rotavirus effects with coincubation with *T. esculentum *extracts	0.94
(b) Survival of H4 cells compared to CIEB cells from rotavirus effects with coincubation with *T. esculentum *extracts	−0.02
(c) Survival of CIEB cells compared to CLAB cells from rotavirus effects with coincubation with *T. esculentum *extracts	0.57
(d) Survival of H4 cells from rotavirus effects with coincubation compared to preincubation with *T. esculentum *extracts	0.03
(e) Survival of CLAB cells from rotavirus effects with coincubation compared to preincubation with *T. esculentum *extracts	0.91
(f) Survival of CIEB cells from rotavirus effects with coincubation compared to preincubation with *T. esculentum *extracts	0.59
(g) Survival from rotavirus effects of, compared to release of nitric oxide, CLAB cells with coincubation with *T. esculentum *extracts	0.43
(h) Survival from rotavirus effects of, compared to release of nitric oxide from H4 cells after coincubation with *T. esculentum *extracts	0.79
(i) Release of NO from H4 cells compared to CLAB cells after coincubation with *T. esculentum *extracts	0.9
(j) Survival from rotavirus effects of, compared to TER, of H4 cells after coincubation with *T. esculentum *extracts for 38 h	−0.39
(k) Survival from rotavirus effects of, compared to TER, of H4 cells after coincubation with *T. esculentum *extracts for 62 h	−0.49
(l) Survival from rotavirus effects of, compared to TER, of H4 cells after coincubation with *T. esculentum *extracts for 67 h	−0.81

## Abbreviations

MCE: *Tylosema esculentum *(marama) cotyledon ethanolic extract
MSCE: *Tylosema esculentum *(marama) seed coat ethanolic extract
MTW: *Tylosema esculentum *(marama) tuber water extract
MCW: *Tylosema esculentum *(marama) cotyledon water extract
MSCW: *Tylosema esculentum *(marama) seed coat water extract
CLAB: Pig small intestine epithelial cell line
CIEB: Bovine small intestine epithelial cell line
H4: Human small intestine epithelial cell line
AZT: Combivir (lamivudine and zidovudine)
RV: Rotavirus
NO: Nitric oxide
TER: Transepithelial resistance.
